# Psychopathology of the General Population Referred by Primary Care Physicians for Urgent Assessment in Psychiatric Hospitals

**Published:** 2016-10

**Authors:** Judith McLenan, Carlo Lazzari, Gail McMillan, Robert Mackie

**Affiliations:** 1 Urgent Referrals Team, Royal Cornhill Hospital, Cornhill Road, Aberdeen, AB25 2ZH United Kingdom.; 2 General Adult Psychiatry, North Essex Partnership University NHS Foundation Trust, The King’s Wood Centre, Colchester, United Kingdom.

**Keywords:** *Depression*, *Personality Disorders*, *Primary Health Care*, *Psychopathology*, *Suicide*

## Abstract

**Objective:** The aim of this study was to evaluate the type, severity and progression of psychiatric pathologies in a sample of 372 outpatients (age range 18–65 years) referred by their primary general practitioners (GPs) to an Urgent Referral Team (URT) based in a psychiatric hospital in Aberdeen, Scotland. This team offers immediate appointments (1- to 7-day delays) for rapid assessments and early interventions to the outpatients referred by their primary family doctors.

**Method:** One-sample t-test and z statistic were used for data analysis. From the total population, a convenience sample of 40 people was selected and assessed to evaluate whether follow-up appointments after the first visit could reduce the severity of suicidal ideation, depression and anxiety in the outpatients seen by the URT. A two-sample t-test and a Wilcoxon signed-rank test were used to assess the variations in the scores during the follow-up visits.

**Results:** We found a statistically significant prevalence of depressive disorders, comorbid with anxiety at first presentation in people who were females, white, never married, living with a partner, not studying and not in paid employment. The common presentation of borderline personality disorder and dysthymia in this population underscores its vulnerability to major socioeconomic challenges.

**Conclusion**: The data confirmed the impact that primary care cooperation with psychiatric hospitals can have on the psychiatric system, and as a reflection, on the population’s mental health and well-being. In fact, active cooperation and early diagnosis and intervention will help detect cases at risk in the general population and reduce admissions into hospitals.

The Urgent Referrals Team (URT) at the Royal Cornhill Hospital in Aberdeen was established in 2005. The team comprises of one or two psychiatrists (a registrar and/or a consultant) and three senior mental health nurses. The catchment area of Aberdeen city includes a population of more than 220,000. The whole city is covered; and the general practitioners (GPs) can refer their patients to the URT if they feel that there is a significant risk to their mental health, existing suicidal ideation or plan, or an important psychopathological problem. GPs that use the URT system commonly perform an initial assessment of depression with the PHQ-9 test (1). In addition, patients are usually seen by the URT from the same day of referral by GPs to a maximum of seven days. Further-more, this population would rarely be seen as inpatients in psychiatric hospitals. Previous research into the prevalence of 2.7% of this population (13.5%) shows moderate to severe degrees of depression (2). Similarly, the WHO collaborative study on psychological disorders in primary health care found that the most common presentations were depression and anxiety (2).

Other authors have also emphasized the importance of collaboration between primary care and mental healthcare providers because it is difficult to manage severe and chronic mental health problems in general practice (3). Regarding the causes that trigger an adjustment disorder with depression, Verhaak et al (4), mentioned family problems (6.1%), material problems (12.8%), work problems (6.5%), and problems in relationships with others than family members (13.9%). This was partly replicated in our study. However, we found that life events could be differentiated into acute (e.g., a split in a relationship, waiting for a court case, etc.), and chronic (e.g., alcohol problems, financial problems, unemployment, or stressed relationships at home) causes. Furthermore, in this research, unreported data seem to suggest that although major life events are adequate to generate reactive depression and anxiety in a healthy person, a vulnerable adult, for example, with long-lasting dysthymia and an emotionally unstable personality may periodically be solicited beyond endurance by minor life events. Other studies also confirm that physicians are more likely to diagnose presentations of depression when it is comorbid with dysthymia than cases where depression was milder (5).

The second part of the research assessed whether the severity of depression at presentation did show some substantial change during the first follow-up visits to the URT. In this case, the sample was assessed with standardized psychiatric tests. Finally, this research aimed to evaluate whether there was a seasonal difference in psychopathologies requiring an urgent assessment. In fact, other authors emphasize a seasonal and monthly variation in the rate of psychiatric service utilization, with summer being prevalent for mood disorders (6).

## Materials and Method

All people presenting for the URT’s services were identified from March 2012 to April 2013. A population of 372 patients was assessed for major psychiatric pathologies and socio-demographic data. These clients were referred by local family doctors (GPs) to the URT when they felt that their clients needed further assessment from a mental health team due to the clients’ risk to themselves and others. The WHO’s ICD-10 diagnostic criteria were used for classification. In this population, the age range was 18 to 65 years. A one-sample t-test and z statistics were used for data analysis. From the total population, a convenience sample of 40 people was also selected to determine whether follow-up appointments after the first visit could reduce the severity in suicidal ideation, depression and anxiety. In this case, a battery of standardized psychiatric tests was used to examine the variations in the parameters observed. In convenience sampling, members of the target population were selected if they satisfied particular criteria such as having a certain diagnosis at presentation (7).

Tests included the Beck’s Depression Inventory (BDI)(8), Beck’s Hopelessness Scale (BHS)(9), Hospital Anxiety and Depression Scale (HADS)(10), Patient Health Questionnaire Version 9 (PHQ-9)(1), Zung Self-Rating Anxiety Scale (SAS)(11), and the Zanarini Rating Scale for Borderline Personality Disorder (ZAN-BPD)(12). Scores on Beck’s Depression Inventory (BDI) were used to assess the severity of depression and existence of suicidal risk (cut-off for the non-case = 17). Finally, a seasonal evaluation was calculated for all the referrals. 

Statistical evaluation comprised of one-sample t-test, and z statistics for proportions were used to examine the data. A two-sample t-test was used to evaluate two observations, whilst a Wilcoxon signed-rank test was used to assess the differences in scores in more than two observations. 

During this study, mixed research methods and triangulations were applied. For ex-ample, an explanatory model was used because the results deriving from quantitative data analysis and psychiatric questionnaires were also explored qualitatively to explain the data (13). At the same time, a triangulation model was also adopted as quantitative data (psychiatric questionnaires), and qualitative data (psychiatric interviews) were collected at the same time (13). The use of multiple questionnaires assessing the same pathologies (and presenting with related items) and the hypothesis of a psychopathological diagnosis gathered through informal assessments characterized a multi-method approach. In this statistical method, several strategies to collect the same data are still compatible with the theoretical approach adopted for the study (14).

Furthermore, data triangulation method was also used, and it was adopted in to decrease the limitations offered by a single source of information or a single questionnaire when the same patient was assessed with questionnaires showing some similarities (15). Finally, an observer or investigator triangulation method was implemented as the same person was often assessed by several health practitioners (e.g., GPs and psychiatrists). However, multiple hypotheses on a diagnosis were shared and confirmed during consecutive investigations (16). 

## Results

***Psychopathology and Socio-demographic Data***

Data refer to the whole population of 372 people. One-sample t-test and z statistics for proportions were used to examine the data (Table 1). A depressive disorder was the prevalent psychiatric presentation with 35% of the cases referred. The socio-demographic data (Table 2) reported no statistically significant difference in the gender of the population that, in 77% of cases, went to the appointment after the referral from their GPs. The population was represented mostly by non-students (99 %), white Scottish (98 %), with a seasonal prevalence of spring referrals from GPs (29%).

***Psychopathology and Severity of Presentation***

A convenience sample of 40 patients was selected for the follow-up study among those who presented with suicidal ideation and were diagnosed as suffering from depression with mixed anxiety and depressive disorder (ICD-10; F41.2), depressive episode (ICD-10; F41.2; F32), recurrent depressive disorder (ICD-10; F41.2; F33), adjustment disorder with mixed anxiety and depressive reaction (ICD-10; F41.2; F43.22), or a persistent mood disorder (ICD-10; F41.2; F34) in emotionally unstable personalities (ICD-10; F41.2; F60.3(.

**Table1:** Psychiatric Diagnosis at First Presentation of General Population Accessing Primary Health Care

	**N (=348)**	**%**	**Significance (two-tailed)**
Depressive disorder	131	35	
Nil psychiatric	46	12	
Substance misuse	51	13	
Schizophrenia, schizotypal, and delusional disorders	28	7.2	
Personality disorders	27	7.2	
Adjustment disorder/Anxiety disorder	50	13	
Bipolar affective disorder	6	1.6	
			t=3.117 (d.f.=9); p=0.01

**Table2:** Sociodemographic Data of General Population with Psychiatric Diagnosis Accessing Primary Health Care

		**N**	**%**	**Significance (two-tailed)**
Gender				z=1.08; p=n.s.
	Male	158	48	
	Female	172	52	
Attendance at the appointment after family doctor’s referral				z=11.86; p<0.05
	Attended	185	77	
	Did not attend or cancelled the appointment	55	23	
Marital status				t=2.51(d.f.=4); p=n.s.
	Married or living with partner	121	38	
	Separated	32	10	
	Divorced	29	9.2	
	Widowed	9	2.8	
	Never married	124	39	
Paid employment in the last 30 days				z=1.2; p=n.s.
	No	165	52	
	Yes	149	47	
Student				z=22.14; p<0.05
	No	337	90	
	Yes	35	9.4	
Seasonal referrals				t=3.91(d.f.=4); p=0.01
	Spring	96	29	
	Summer	77	23	
	Autumn	79	23	
	Winter	78	23	
First language				z=17.06; p<0.05
	English	305	81	
	Other	71	19	
Ethnicity				z=24.13; p<0.05
	White	306	98	
	Others	5	1.6	

**Suicide Risk and Depression:** The one-sample t-test revealed that the mean value for BDI was indicative of a moderate to severe depression at presentation (t = 4.107, d.f. = 12, p =0.001, mean scores at 95% CI = 22.597–35.249). The results revealed that the Wilcoxon W was not statistically significant (W = 3.0, alpha = 0.05; p = 0.346) for the difference in the severity of depression at first assessment and first follow-up visits. The t-test for two independent samples (first assessment and first-week follow-up) did not produce a statistically significant difference (t = 0.727, d.f. = 1; two-tailed p = 0.5). Therefore, the suicidal risk and severity of depression remained active during the first assessment and persisted intensely and unchanged in the follow-up visit after about one week, although scores on the BDI tended to be lower (initial assessment: mean = 23.11, SD = ±13.87; first follow-up visit: mean = 19.66; S.D. = ±5.85) (Table 1). 

**Anxiety:** The Zung Self-Rating Assessment Scale (mean = 45.45±6.7) indicated a level of anxiety above the cut-off of 36 for Generalized Anxiety Disorder. A severe lev-el of anxiety during the first presentation was confirmed by the anxiety sub-scale of the HAD (mean = 14.85±3.5). However, anxiety levels tended to decrease during follow-up visits. At first presentation, high levels of depression (BDI) were positively correlated with elevated levels of anxiety (Zung) (r = 0.75; t = 2.77; p = 0.01). 

**Emotionally Unstable Personality Disorder (EUPD):** EUPD was also a frequent presentation (67.5%), and the values on ZAN-BPD (mean = 5.8±2.8) confirmed that this personality disorder can be comorbid with depression and dysthymia, with higher values in the ZAN-BPD tending to correlate with higher values on the BDI depression scale (r= 0.72; t = 2.54; p = 0.02).

**Seasonal variations:** For what concerns the seasonal variation of referrals, there was a statistically significant difference in the months of the year, with an average of 27.5 (±5.01) new referrals per month. A statistically significant difference was also detected in the season of referrals (t = 3.91; d.f. = 4; p = 0.01) with spring being the period with the highest number of referrals (29%), while the other months were at 23% with respect to frequency (Table 2).

## Discussion

We found that the usual psychopathology that GPs refer to secondary care is represent-ed by a depressive disorder accompanied by recent stressors and a high risk to the self, usually expressed with suicidal ideation and intent. Moreover, data revealed that depression and anxiety tend to be elevated and co-morbid during the initial assessment from the URT. The patient commonly referred by GPs to secondary care was also a white female never married, unemployed, and with no formal education. 

For what concerns the psychopathology of the most common presentation, this re-search highlights the repercussion on the health system represented by people with an emotionally unstable personality disorder comorbid with dysthymia and suicidal ideation possibly triggered by recent life events. The data confirm the impact that primary care can have on the mental health system and, as a reflection, on the population’s mental health. As underscored by other authors, improved mental health leads to enhanced physical health and lower mortality (17). As this research proves, there is a high percentage of visitors to the URT on the part of patients referred by GPs. Therefore, this leads to a higher prevention of potential suicides in the general population by using a quick referral system and follow-up similar to the one adopted in this study.

The GPs who participated in this project reported that they received support from the URT when there was some concern about the mental health in their patients. In fact, some authors have found that GPs spend 3 to 4 minutes more with patients who have a comorbid mental health and medical condition than with patients with no psychiatric diagnosis (18). It is expected that the number of people in England who will present a mental health problem will be 9.88 million in 2026, respecting an increase in the population (19). Moreover, 35% of people with depression and 51% of people with anxiety are not in contact with primary or secondary care services (19). 

## Limitations

This study carries some weaknesses. In fact, there were no data reporting the rate of re-lapse once a patient was discharged from the URT to GPs or to a community mental health team for follow-up. In addition, further research is needed to investigate the intrapersonal and interpersonal factors during follow-up visits that might be beneficial for people with depression and suicidal ideation. Finally, although the diagnostic criteria were standardized, the mental health practitioners involved in this study did not always have the same theoretical, educational and clinical background. This could influence the validity of the study and the diagnoses adopted, calling for further attention when con-ducting similar studies. 

## Conclusion

Family physicians often represent the healthcare providers who deal for the first time with psychiatric crises, the first assessment, or the first referral of people who have never contacted secondary care and mental-health services. In addition, early intervention with this population would reduce the cost of lost employment due to poor mental health (19). Thus, timely involvement, like that offered by the URT, also carries a secondary and economic benefit by reducing clients’ absences from jobs due to a mental health crisis. At the same time, a reduction of unnecessary admissions into a psychiatric hospital is also foreseeable. 
